# *FGFR3-TACC3* fusion in solid tumors: mini review

**DOI:** 10.18632/oncotarget.10482

**Published:** 2016-07-07

**Authors:** Ricardo Costa, Benedito A. Carneiro, Timothy Taxter, Fabio A. Tavora, Aparna Kalyan, Sachin A. Pai, Young Kwang Chae, Francis J. Giles

**Affiliations:** ^1^ Developmental Therapeutics Program, Division of Hematology and Oncology, Feinberg School of Medicine, Northwestern University, Chicago, Illinois, USA; ^2^ Robert H. Lurie Comprehensive Cancer Center of Northwestern University, Chicago, Illinois, USA; ^3^ Department of Pathology, Feinberg School of Medicine, Northwestern University, Chicago, Illinois, USA; ^4^ Department of Pathology, Messejana Heart and Lung Hospital, Fortaleza, Brazil

**Keywords:** FGFR3-TACC3 fusion, non-small cell lung cancer, phosphatidylinositol 3-Kinase (PI3K), aneuploidy, glioblastoma multiforme

## Abstract

Fibroblast growth factor receptors (FGFR) are transmembrane kinase proteins with growing importance in cancer biology given the frequency of molecular alterations and vast interface with multiple other signaling pathways. Furthermore, numerous FGFR inhibitors in clinical development demonstrate the expanding therapeutic relevance of this pathway. Indeed, results from early phase clinical trials already indicate that a subset of patients with advanced tumors derive benefit from FGFR targeted therapies. *FGFR* gene aberrations and *FGFR* gene rearrangements are relatively rare in solid malignancies. The recently described *FGFR3-TACC3* fusion protein has a constitutively active tyrosine kinase domain and promotes aneuploidy. We summarize the prevalence data on *FGFR3-TACC3* fusions among different histological tumor types and the preliminary evidence that this rearrangement represents a targetable molecular aberration in some patients with solid tumors.

## INTRODUCTION

The growing knowledge base of tumor genomics has led to never seen advances in the field of medical oncology. [1] The evolving molecularly targeted treatments of late-stage melanoma, gastro-intestinal stromal tumors, and non-small-cell lung cancer (NSCLC) exemplify these advances. [2–4]

**Figure 1:**
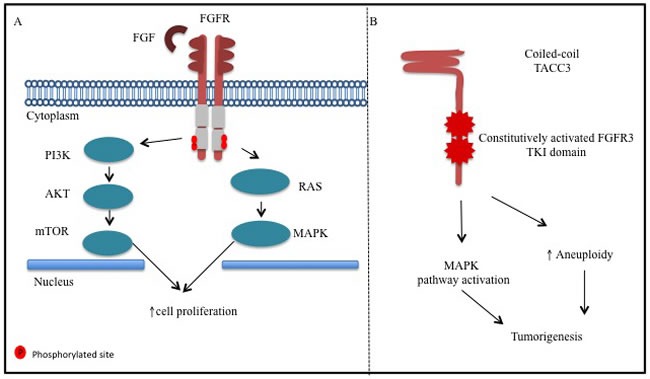
**A**. Fibroblast growth factor receptor (FGFR) has intra-cellular tyrosine kinase activity triggered by fibroblast growth factor (FGF) ligand. Its activation leads to FGFR transphosphorylation and activation of protein of *ras* oncogene (RAS)/mitogen-activated protein kinase (MAPK) and phosphatidylinositol 3-Kinase (PI3K) signaling pathways. **B**. Fibroblast growth factor receptor3-transforming acidic coiled-coil containing protein 3(FGFR3-TACC3) fusion protein harbors constitutively activated tyrosine kinase domain, which activates mitogen-activated protein kinase (MAPK) pathway. Also, FGFR3-TACC3 localizes to mitotic spindle poles, induces mitotic, chromosomal segregation defects and triggers aneuploidy.

The fibroblast growth factor receptor (FGFR) family consists of four subtypes of transmembrane tyrosine kinase receptors that play an important role in cell growth, differentiation and angiogenesis *via* binding of up to 22 known different FGF family ligands. [5] Upon FGFR activation through dimerization of receptor monomers and transphosphorylation of kinase domain loop tyrosine residues cytoplasmatic downstream molecules contribute to carcinogenic events mediated by PI3K/AKT, STAT and RAS/MAPK pathways (Figure 1A). [5, 6]

Anomalous signaling through FGFR can occur through overexpression of receptors, activating mutations, gene amplification, or by FGFR-containing translocations in wide array of solid tumors as discussed below. Most importantly, these aberrations can represent important therapeutic targets in solid tumors and have supported the clinical development of several FGFR inhibitors and anti-FGFR drug conjugate (ADC) antibodies (Tables 3, 4, 5, 6, 7, 8, 9). Herein we review the current literature and describe the cancer pathobiology, the prevalence, the pre-clinical, and the clinical data supporting drug development targeting the recently described *FGFR3-TACC3* fusion.

## FGFR ABERRATIONS

*FGFR* alterations relatively rare and were present in ~7% of a cohort of 4853 tumor samples including 47 different histological types. [7] When limiting to histologies with at least 75 samples analyzed, the frequencies of *FGFR* aberrations were higher among urothelial (31.7%), breast (17.4%), endometrial (11.3%), and endometrial/ovarian carcinomas (8.1%). [7] The majority of *FGFR* genomic alterations were *FGFR* amplifications and mutations (92%).

Evidence from early phase clinical trials support that *FGFR* aberrations can represent targetable events in solid tumors. In a phase I trial, twenty one patients with refractory squamous non-small cell lung cancer (sqNSCLC) harboring *FGFR1*-amplification were treated with the small molecule FGFR inhibitor BGJ398 (100 or 125 mg once daily in 28-day cycles). [8] Of 17 evaluable patients, 4 had radiologic tumor reduction and 3 had stable disease indicating that a subset of patients with FGFR pathway aberrations indeed benefits for FGFR targeted therapy. In another phase I trial BGJ398 also showed evidence of clinical activity in patients with solid tumors harboring *FGFR* aberrations, including 4 of 5 patients with urothelial cell carcinomas (4 of which originated in the bladder) with *FGFR3*-activating mutations. Additionally, two patients with *FGFR1*-amplified sqNSCLC achieved confirmed partial response. Tumor reductions were also observed in cholangiocarcinoma with an *FGFR2* gene fusion, and *FGFR1*-amplified breast cancer. [9] In summary, given the rarity of *FGFR* aberrations the preliminary evidence of antitumor activity of FGFR targeted therapies comes from subpopulations of small early phase clinical trials. A small number of partial responses to FGFR targeted therapies have been documented among patients with sqNSCLC harboring *FGFR1* amplification (BGJ398), cholangiocarcinoma harboring *FGFR2* translocations (BGJ398), glioblastoma positive for *FGFR3* translocation (JNJ-42756493), bladder cancer harboring *FGFR3* mutations and translocations are sensitive to targeted therapies (JNJ-42756493 and BGJ398). [8–11]

## *FGFR3-TACC3* TRANSLOCATION

Fusions have been described in the *FGFR1-3* genes with multiple partners (i.e., *TACC1*, *TACC2*, *TACC3*, *BAIAP2L1*, *BICC1*, *NPM1*, *PPAPDC1A*, *AFF3*, *SLC45A3* and *AHCYL1*) in a wide spectrum of tumors (i.e., cholangiocarcinoma, breast, and prostate cancer, sqNSCLC, gastric adenocarcinoma, colorectal adenocarcinoma, carcinoma of unknown primary and glioblastoma). [7, 12–15]

Other rare *FGFR* fusions described included *FGFR2-TACC3* (1), *FGFR2-NPM1*(3), *FGFR2-TACC2*(2), *FGFR2-BICC1*(2), *FGFR2-C10orf68*(1), *FGFR3-JAKMIP1*(1), *FGFR2-KIAA1598*(1), *FGFR2-NCALD*(1), *FGFR2-NOL4*(1), *FGFR1-NTM*(1), *FGFR2-PPAPDCA*(1), *FGFR3-TNIP2*(1), and *FGFR3-WHSC1*(1). Of note no *FGFR4* gene fusion was observed in this large series. [7] Preliminary pre-clinical data support that these fusions represent important therapeutic targets. For instance stable cell lines harboring *FGFR3-BAIAP2L1*, *FGFR3-TACC3*, and *FGFR2-CCDC6* fusions showed expression of active FGFR fusion kinases and activation of downstream mitogen-activated protein kinase ERK1/2 and the transcription factor STAT1. [12] The presence of *FGFR* fusions (*FGFR3-BAIAP2L1*) not only enhanced tumor cell proliferation, but also led to significant sensitivity to small kinase inhibitors (PD173074) in pre-clinical cellular and xenograft bladder cancer models, in contrast with *FGFR3* mutant cell lines, which were not sensitive to kinase inhibition. [12] *FGFR1-TACC1* fusion targeted therapy also showed anti-tumor effects in pre-clinical GBM model. [13] Cholangiocarcinoma mice model harboring *FGFR2-AHCYL1*, *FGFR2-CCDC6*, and *FGFR2-BICC1* was sensitive to treatment with FGFR kinase inhibitors BGJ398 and PD173074. [16, 17]

*TACC3* gene has been identified as an important partner of these *FGFR* fusions and associated with the pathogenesis of several solid tumors. [18–20] *TACC3* (transforming acidic coiled-coil containing protein 3) belongs to the *TACC* gene family, which also includes *TACC1* and *TACC2*. TACC3 protein has a coiled-coil domain at the C terminus, known as the TACC domain, which promotes stability and organization of mitotic spindle. [21]

*FGFR3-TACC3* fusions were first described in glioblastoma multiforme (GBM) and bladder urothelial tumors. [13, 15] Subsequent manuscripts underscored the low frequency of *FGFR3-TACC3* gene fusions across different tumor types.

Among the 4853 tumors samples analyzed by Helsten et al. only 28 exhibited *FGFR* gene fusions, and 14 samples had *FGFR3-TACC3* fusions. Accompanying genomic aberrations observed primarily affected *TP53* tumor suppressor gene, AKT/mTOR/PTEN pathway and cell cycle control genes (i.e., *CNNE1*, *CDK2* and *4*) (Table 1). [7]

**Table 1:** Co-existing genomic aberrations among 14 *FGFR3-TACC3* fusion cases.[7]

Histology	Cell cycle	PI3Kinase	TP53	DNA repair	Transcription/Histone methylation	Growth factor receptor	RAS/RAF/MAPK	Other
CUP	*CDK4*-amplification							*MDM2-amp ARID1A-A343_A348>A*
Cervical cancer		*PIK3CA-E545K*						
Cervical cancer			*TP53-R248Q*	ATM-V2115fs*5				
Endometrial adenocarcinoma		*PIK3CA-E365K*	TP53-W91*			FLT3-E978*		*KMT2A*-complex rearrange
Gallbladder carcinoma	*CCNE1*-amplification		TP53-C141*		*MYC*-amplification			*MCL1*-amplification
Glioma			*TP53-E258K*					NF1 Y2285fs*5, Q270* MCL1-amplification *NFKBIA*-amplification *PTPN11-E76K*
Glioma	*CDK4*-amplification	*PTEN-splice site 209+1delGT*						*MDM2*-amplification
NSCLC Not specified	*CDKN2A*-loss							*MDM2*-amplification
Pancreatic exocrine carcinoma			*TP53-I195F*	ATM-R805*	*MYC*-amplification			*SMAD4*-loss
Renal cell carcinoma	*CDKN2A/B*-loss		TP53-R196*		*MYC*-amplification			*TOP1*-amplification *SRC*-amplification *AURKA*-amplification *VHL-P25L*
Urothelial carcinoma	*CCND1*-amplification	*PIK3CA-H1047R*	*TP53-R280T*					
Urothelial carcinoma			*TP53-K132N*					*JUN*-amplification *IRS2*-amplification *MCL1*-amplification
Urothelial carcinoma	*CCND1*-amplification *CDKN2A/B*-loss	PIK3R2-E543*				*ERBB2* amplification	*MAP3K1*-truncation, exon 15	*MDM2*-amplification *NF1-I1351M*
Urothelial carcinoma	*CDKN2A/B*-loss							*IRS2*-amplification *MDM2*-amplification

Abbreviations: Non-small cell lung cancer (NSCLC), carcinoma of unknown primary (CUP), mouse double minute 2 homolog (MDM2), cyclin E1 gene (CCNE1), cyclin D1 gene(CCND1), cyclin dependent kinase(CDK), cyclin dependent kinase inhibitor (CDKN), phosphatidylinositol 3-Kinase (PI3K), Erb-B2 receptor tyrosine kinase 2 (ERBB2), tumor protein P53(TP53), phosphatase and tensin homolog(PTEN), V-Myc Avian Myelocytomatosis viral oncogene homolog (MYC), ataxia telangiectasia mutated(ATM), mitogen-activated protein kinase 1 (MAP3K1), AT rich interactive domain 1A (ARID1A), lysine (K)-specific methyltransferase 2A(KMT2A), myeloid cell leukemia 1(MCL1), neurofibromin 1(NF1), Nuclear Factor Of Kappa Light Polypeptide Gene Enhancer In B-Cells Inhibitor, alpha (NFKBIA), protein tyrosine phosphatase, non-Receptor Type 11(PTPN11), SMAD family member 4(SMAD4), topoisomerase (DNA) I(TOP1), SRC proto-oncogene(SRC), aurora kinase A(AURKA), von hippel-lindau tumor suppressor (VHL), Jun proto-oncogene(JUN), insulin receptor substrate 2(IRS2).

**Figure 2:**
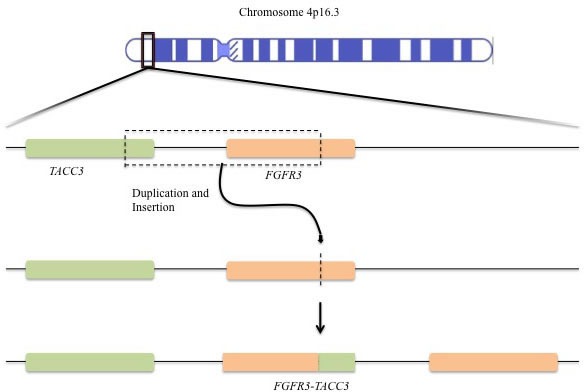
*FGFR3-TACC3* gene fusion Tandem duplication and insertion leads the fusion of the tyrosine kinase domain of *FGFR3* to the *TACC* domain of *TACC3.*

*FGFR3-TACC3* fusions are formed by rare intrachromosomal rearrangements located within 150 kb of the *FGFR3* gene on chromosome 4p16 (Figure 2). [13] It has been proposed that the fusion, which occurs *via* a tandem duplication event, leads to loss of an miR-99a binding site within the 3′-untranslated region (3′- UTR) of *FGFR3*, releasing *FGFR3* signaling from miR-99a-dependent inhibition and enhancing tumor progression relative to wild type *FGFR3*. [22] Given the close proximity of the *FGFR3* and *TACC3* genes, identification of the fusion by FISH is technically challenging. [23] Therefore the most common methods have involved transcriptomic analysis by RNA-seq and RT-PCR in addition to whole-genome sequencing. [7, 13, 23] Di Stefano et al. have also utilized immunostaining of the N terminus of FGFR3 and found uniform overexpression of FGFR3 in a subset of glioblastoma multiforme (GBM) cases with *FGRF3-TACC* fusions that were identified by RT-PCR. [23] Those findings demonstrate that detection of FGFR3 amplification by IHC or IF may serve as a method to screen for *FGRF3-TACC* fusions which is clinically relevant since RNA sequencing is not common practice. [24] The fusion protein exhibits constitutive kinase activity, induces mitotic, chromosomal segregation defects and triggers aneuploidy (Figure 1B). [13] The presence of the TACC coiled-coil domain increases the activity of FGFR3 through more promiscuous constitutive phosphorylation of tyrosine kinase residues within the FGFR3 protein, and preferential MAPK kinase activation (Figure 1A). [25] Moreover, preclinical and clinical results have shown that FGFR inhibitors can block the tyrosine kinase activity of this fusion protein.

**Table 2:** Cross-sectional studies and case series reporting positive *FGFR3-TACC3* fusions (excluding TCGA dataset samples)

Authors	Tumor type	Number of cases analyzed	Number of cases harboring *FGFR3-TACC3* fusion	*FGFR3* breakpoint	*TACC3* breakpoint	Comments
Helsten *et al.*[7]	Urothelial carcinoma	126	4	NR	NR	Organ site was not specified
Williams *et al.*[15]	Bladder carcinoma	32	2	Exon 18	Exon 13	
Singh *et al.*[13]	GBM	97	2	Exon 17	Intron 7	
Di Stefano *et al.*[23]	Gliomas	795	20	Exon 17,18	Exon 4, 5, 6, 8, 10, 11	17 patients had GBM and 3 patients grade III or II gliomas
Parker *et al.* [22]	GBM	48	4	--	--	
Bao Z. *et al* [27]	GBM	59	3	Exon 17	Exons 8,10,11	
Helsten *et al*. [7]	NSCLC (subtype not specified)	675	1	NR	NR	NSCLC subtype was not specified
Capelletti *et al*.[30]	Adenocarcinoma of the lung	576	3	Exon 17	Exon 4, 8, 11	
Wang *et al.* [31]	NSCLC (6 adenocarcinomas 9 sqNSCLC)	1328	15	Exon 17,18	Exon 5,8,10,11	6 cases of adenocarcinoma and 9 cases of SCC; FGFR3-TACC3 correlated independently with tumor size > 3 cm
Kim *et al*.[32]	sqNSCLC	104	2	Exon 17, 18	Exon 8,9	Author also found 4 more cases at the TGCA dataset
Majewski *et al.*[33]	sqNSCLC	95	2	Exon 18	Exon 10	Fusion was identified in 2 SCC cases
Carneiro *et al*.[36]	Cervical cancer: SSC and adenosquamous cell carcinoma,	--	3	Intron 17,18	Intron 7,10	
Xiang *et al.* [37]	Cervical cancer: SCC, adenocarcinoma, adenosquamous, small cell carcinoma	285	11	--	--	All early stage tumors
Helsten *et al.* [7]	Cervical adenocarcinoma and cervical carcinoma not specified	48	2	Intron 17	Intron 7	Fusion reported for carcinoma NOS
Helsten *et al.* [7]	Carcinoma of unknown primary	267	1	NR	NR	
Helsten *et al.* [7]	Endometrial Carcinoma	80	1	NR	NR	
Helsten *et al*.[7]	Gallbladder carcinoma	47	1	NR	NR	
Helsten *et al.* [7]	Glioma	144	1	NR	NR	
Helsten *et al.* [7]	Pancreatic exocrine tumor	172	1	Exon 18	Intron 10	
Helsten *et al.* [7]	Renal cell carcinoma	87	1	NR	NR	
Parish *et al*.[39]	Solid tumors	391	1	NR	NR	

Non-small cell lung cancer (NSCLC), squamous non-small cell lung cancer (sqNSCLC), glioblastoma multiforme (GBM), fibroblast growth factor receptor 3-transforming acidic coiled-coil containing protein 3(FGFR3-TACC3), Not reported (NR), squamous cell carcinoma (SCC), The Cancer Genome Atlas (TCGA).

Treatment of nasopharyngeal carcinoma cells carrying the *FGFR3-TACC3* fusion with FGFR inhibitor PD173074 inhibited cell proliferation. [26] RT4 urothelial carcinoma line harboring *FGFR3-TACC3* fusion also exhibited sensitivity to this same FGFR inhibitor in a xenograft model. [12] Similar results were seen in GBM *in vivo* and *in vitro* models carrying the *FGFR3-TACC3* fusions, which was not only associated oncogenic transformation but also exhibited significant sensitivity to FGFR inhibitor JNJ-42756493. [13, 22, 23]

The clinical relevance of *FGFR3-TACC3* has been underscored by preliminary results from clinical studies and case reports of tumor responses to the treatment with FGFR inhibitors. For instance, the phase I trial with FGFR inhibitor JNJ-42756493 including 65 patients with advanced solid tumors included 4 patients with *FGFR3-TACC3* translocation. [10] Three partial responses (two confirmed and one unconfirmed) were seen among 3 patients with urothelial, two of which cancer harbored *FGFR3-TACC3* fusion. One of these patients stayed on treatment for about 10 months. Another confirmed partial response was observed in a patient with glioblastoma with *FGFR3-TACC3*. Tumor shrinkage was also seen in a patient with adrenal carcinoma with *FGFR3-TACC3/FGFR2- CCDC6* (*FGFR3-TACC3* being the predominant translocation), who received treatment for 10 months before disease progression. In agreement with preclinical results, these results suggest that *FGFR3-TACC3* fusion is indeed an actionable therapeutic target.

## PREVALENCE OF FGFR3-TACC3 FUSION IN SOLID TUMORS

### Gliomas

The first report of this fusion emerged from a small subset of patients with GBM with 2 out of 97 samples harboring *FGFR3-TACC3*. [13] A subsequent study with 795 cases of gliomas (584 GBMs and 211 grade II-III gliomas) described 17 cases (2.9%) of *FGFR3-TACC3* genomic fusions among the GBM group, and 3 cases among lower grade gliomas. [23] Amplicons ranged from 928 bp (for *FGFR3*exon18-*TACC3*exon13) to 1,706 bp (for *FGFR3*ex18-TACC3ex4). All tested *FGFR3-TACC3* positive tumors showed strong expression of FGFR3 by tumor cells on immunohistochemistry (IHC). Epidermal growth factor receptor gene (*EGFR*) amplification showed significant negative correlation with *FGFR3-TACC3* status; *CDK4* and *MDM2* amplification had significant positive correlation with *FGFR3-TACC3* (*CDK4* amplification was seen in 7/16 *FGFR3-TACC3* positive cases). No statistically significant correlation was seen between *FGFR3-TACC3* fusions and other genetic and epigenetic alterations (*CDKN2A* deletion, *TERT* promoter mutations, gain of chromosome 7p, loss of chromosome 10q, and methylation of the *MGMT* promoter). Parker et al. also reported higher frequency of *FGFR3-TACC3* fusions in GBMs when compared to gliomas. [22] Four out of 48 (8.3%) GBMs tested were positive for this fusion, whereas none of the 43 low-grade glioma samples showed this translocation. In addition, 2 out 157 GBM (1.2%) and 2 out of 461 low grade gliomas (0.4%) samples from The Cancer Genome Atlas (TCGA) dataset tested positive for *FGFR3-TACC3* fusions which reinforced the notion that this fusion is more common in high grade gliomas. [14] In another series 3 out of 59 patients with primary GBM harbored this fusion gene. [27] Wu et al. reported 2 cases of GBM harboring *FGFR3-TACC3* fusion from the TGCA database. [12] In summary, it is estimated that 1.2-8.3% of GBMs will carry this translocation.

### Urothelial cancer

The TCGA project reported a comprehensive genomic analysis of 131 high-grade muscle invasive urothelial bladder carcinomas including gene fusions. Three cases of tumors with *FGFR3-TACC3* fusion were reported (~2.3%). The breakpoints were in intron 10 of *TACC3* and intron 16 (2 cases) or exon 17 (1 case) of *FGFR3*. [28] Two cases among 99 samples analyzed showed two distinct *FGFR3-TACC3* genomic fusions; first, intron 17 of *FGFR3* with intron 10 of *TACC3* resulting in exon 17 of FGFR3 being spliced 5′ to exon 11 of *TACC3* in the fuse mRNA; second, intron 17 of *FGFR3* with exon 4 of *TACC3* at the gene level and in the fused mRNA, exon 17 and fragment of intron 17 in *FGFR3*, and fragment of exon 4 in *TACC3* were merged into a novel exon. [29] Analysis of 250 samples of bladder urothelial carcinoma by RNA-seq detected *FGFR3-TACC3* in 5 specimens (2%). [14] Helsten et al. described 126 cases of urothelial carcinomas and 4 cases (3%) of *FGFR3-TACC3* translocations were observed. [7] Williams et al. reported results of analysis of 2 tumor samples positive for *FGFR3* exon 18 and *TACC3* exon 13 fusions among 32 selected bladder carcinoma samples. [15] Taken together, a total of 14 cases of urothelial carcinoma carrying *FGFR3-TACC3* translocation have been described with an estimated incidence of 2.6% among the 539 cases described above.

### Non-small cell lung cancer

RNA sequencing of 492 sqNSCLC from the TCGA showed only 3 cases of *FGFR3-TACC3* fusions; none of 513 cases of adenocarcinomas harbored this translocation. [14] Another study describing genomic analysis of 675 cases of NSCLC showed only one case of *FGFR3-TACC3* fusion (breakpoint not specified), this was associated with existing *CDKN2A*-loss and *MDM2* amplification. [7] Wu et al. reported through analysis of the TGCA dataset 4 cases of sqNSCLC harboring *FGFR3-TACC3* fusion. [12] Capelletti et al. identified 3 out 576 patients with adenocarcinoma of the lung harboring *FGFR3-TACC3*. [30] Fusion variants identified included exon 17 *FGFR3* to exon exon 8 of *TACC3*; exon 17 of *FGFR3* and exon 11 of *TACC3*; and exon 17 of *FGFR3* to exon 4 *TACC3* resulting in an overall prevalence of 0.5%. An additional analysis of 1,328 NSCLC samples revealed 15 *FGFR3-TACC3* fusion variants identified through RT-PCR [31]. Histological distribution was as follows: 6/1016 (0.6%) adenocarcinomas and 9/312 (2.9%) squamous cell carcinoma of the lung suggesting the *FGFR3-TACC3* fusions are more frequent in sqNSCLC. *FGFR3-TACC3* fusion correlated with positive smoking history and male gender in univariate analysis. Tumor size > 3cm correlated with *FGFR3* fusions independently in multivariate modeling. *FGFR3-TACC3* fusions were distributed as follows: E18:E11 2 cases, E17:E11 9 cases, E17:E8 1 case, E17:E5 1 case, and E17:E10 2 cases.

Another cohort of patients from Korea was analyzed and only 2 cases of *FGFR3-TACC3* fusion were detected (E17:E8 and E18:E9). The same author reviewed the samples of the TCGA database and found 4 out of 178 samples positive for this fusion. [32] Two more cases of *FGFR3* E18 fused with *TACC3* E10 were reported in sqNSCLC. [33] The TCGA dataset reported genomic analysis through RT-PCR of 230 previously untreated adenocarcinomas of the lung and 178 previously untreated sqNSCLC; *FGFR* fusions were not reported in either series. [34, 35] In summary, *FGFR3-TACC3* fusions are rare in NSCLC but seem to be more common in sqNSCLC histological type with an estimated prevalence consistently lower than 2.9%.

### Cervical cancer

Three cases of *TACC3-FGFR3* translocations break points in patients with advanced cervical carcinoma have been described with early evidence of clinical benefit from FGFR targeted treatment. [36] One of the cases was a metastatic recurrent to the lung adenosquamous carcinoma of the cervix. The metastatic tumor showed *FGFR3-TACC3* fusion (break point intron 17 and *TACC3* intron 10). Associated genomic aberrations were: *AKT1* missense mutation, *mTOR* point mutation, *ATRX* truncating nonsense mutation. The patient was treated with FGFR targeted therapy achieving stable disease for 4 cycles. In the second case, the patient had well differentiated squamous cell carcinoma of the cervix stage. Tumor tissue from the original biopsy showed *FGFR3-TACC3* fusion (break points *FGFR3* intron 18 and *TACC3* intron 7) as well as *BRAF 3′* tandem duplication, activating *PIK3CA* missense mutation, *CDNK2A* loss, and activating missense mutations in *KRAS* and *HRAS*. The third case was a squamous cell carcinoma of the cervix in which the following genomic aberrations were identified on hysterectomy specimen: *FGFR3-TACC3* fusion (breakpoints at *FGFR3* intron 17 and *TACC* intron 10) and partial loss of *STK11*, and *RB1* loss.

Xiang et al. performed transcriptomic (RNA) followed by cDNA analysis of 285 cases of early stage carcinoma of the cervix; 11 cases of *FGFR3-TACC3* translocations were described (3.9%) with 4 variants [*FGFR3* (1_758) fused with *TACC3* (549_838), *FGFR3*(1_758) fused with *TACC3*(648_838), *FGFR3* (1_758) fused with *TACC3* (648_838), and the most frequent variant *FGFR3* (1_768) fused with *TACC3*(538_838)] [37]. There were four cases of squamous cell carcinoma, 3 cases of adenocarcinoma 3 cases of adenosquamous, and 1 small cell carcinoma. Other sporadic cases of *FGFR3-TACC3* translocation in cervical carcinoma have been reported as part of genomic aberrations analysis of solid tumors: *FGFR3-TACC3* fusion (intron 17-intron 7) one case of cervical carcinoma. [7]

### Head and neck cancer

Among 411 head and neck SCC tumor samples analyzed using RNA sequencing data through the TGCA only 2 harbored *FGFR3-TACC3* fusion. [14] Wu et al. reported two additional cases of head and neck cancer harboring this fusion. [12] An additional analysis of 279 head and neck SCC tumor samples from the TGCA dataset (172 oral cavity, 33 oropharynx, 72 laryngeal tumors) and cases of *FGFR3-TACC3* were appreciated in two of the HPV+ samples. [38]

### Gastrointestinal malignancies

*FGFR3-TACC3* fusions are rare event as illustrated by analysis RNA sequencing of 856 tumor samples [hepatocellular carcinoma (194), colon (286), rectum (91), and gastric adenocarcinomas (285)] ; in which no tumor showed this translocation. [14]

### Other malignancies

Two studies analyzed a total of 1594 cases of breast cancer no *FGFR3-TACC3* fusion was reported. [7, 14] Single cases of carcinoma of unknown primary, endometrial carcinoma, renal cell carcinoma, gallbladder, papillary kidney tumor, and prostate adenocarcinoma harboring the *FGFR3-TACC3* fusion have been described. [7, 14]

**Table 3:** Ongoing selected clinical trials targeting the FGFR pathway in solid tumor and hematologic malignancies

Drug	Mechanism of action	Phase	Study population	Clinicaltrials.gov Identification
ARQ 087	FGFR1-3 TKI	1/2	Solid tumors with FGFR genetic alterations, including intrahepatic cholangiocarcinoma with *FGFR2* gene fusion	NCT01752920
AZD4547	FGFR1-3 TKI	2	*FGFR1* or *FGFR2* amplified breast, squamous lung and stomach cancer	NCT01795768
AZD4547	FGFR1-3 TKI	1	In the dose expansion phase participant must have solid tumors with *FGFR1* and/or *FGFR2* gene amplified sqNSCLC, *FGFR1* gene low & high amplified or gastric adenocarcinoma, including the lower esophagus/gastro-esophageal junction, *FGFR2* gene low & high amplified	NCT00979134
AZD4547	FGFR1-3 TKI	2	Advanced Gastric Adenocarcinoma (Including adenocarcinoma of the lower third of the esophagus or the gastro-esophageal junction) with FGFR2 polysomy or gene amplification.	NCT01457846
AZD4547	FGFR1-3 TKI	2a	ER+ breast cancer patients With FGFR1 polysomy or gene amplification who have progressed following treatment with prior endocrine therapy	NCT01202591
AZD4547	FGFR1-3 TKI	2a	Refractory metastatic ER+ breast cancer	NCT01791985
AZD4547	FGFR1-3 TKI	1	Japanese patients with advanced solid tumors	NCT01213160
BAY1163877	FGFR1-3 TKI	1	In the dose expansion cohort patient must have histological or cytological sqNSCLC, lung adenocarcinoma, head and neck cancer or bladder cancer	NCT01976741
BAY1187982	Anti-FGFR2 antibody drug conjugate	1	Advanced solid tumors known to express FGFR2	NCT02368951
BAY1179470	Anti FGFR2 antibody	1	Refractory solid tumors with at least moderate FGFR2 expression in the tumor tissue from archival samples is confirmed	NCT01881217
BGJ398	FGFR1-3 TKI	2a	*FGFR1-3* translocated, mutated, or amplified squamous cell carcinoma of the head and neck	NCT02706691
BGJ398	FGFR1-3 TKI	2	Solid tumor (except with a primary diagnosis of UC, cholangiocarcinoma, endometrial cancer, and GBM) or hematologic malignancies with *FGFR* genetic alteration	NCT02160041
BGJ398	FGFR1-3 TKI	1	Advanced solid tumors with *FGFR1* or *FGFR2* amplification or *FGFR3* mutation, for which no further effective standard anticancer treatment exists or UC with *FGFR3* mutations or gene fusions progressing after platinum-based chemotherapy or intolerant to platinum therapy or for whom platinum is contraindicated	NCT01004224
BGJ398 combined with chemotherapy	FGFR1-3 TKI	1b/2	Advanced and metastatic pancreatic cancer	NCT02575508

Abbreviations: Fibroblast growth factor (FGF), fibroblast growth factor receptor (FGFR), not reported (NR), estrogen receptor positive (ER+), tyrosine kinase inhibitor (TKI), bacillus calmette-Guerin (BCG), small cell lung cancer (SCLC), Non-small cell lung cancer (NSCLC), squamous non-small cell lung cancer (sqNSCLC), pilot study (PS), glioblastoma multiforme (GBM), hepatocellular carcinoma (HCC), Malignant pleural mesothelioma (MPM), immunohistochemistry (IHC), urothelial carcinoma (UC).www.clinicaltrials.gov accessed on April 22^nd^ 2016.

**Table 4:** Ongoing selected clinical trials targeting the FGFR pathway in solid tumor and hematologic malignancies continued

Drug	Mechanism of action	Phase	Study population	Clinicaltrials.gov Identification
BGJ398	FGFR1-3 TKI	2	Advanced or metastatic cholangiocarcinoma with FGFR2 Gene Fusions or Other FGFR genetic alterations who failed or are intolerant to platinum-based Chemotherapy	NCT02150967
BGJ398/BYL719	FGFR1-3 TKI	1b	Refractory solid tumor with PIK3CA mutations in all patients in dose escalation and expansion with or without documented genetic alterations in FGFR depending upon dose expansion cohort	NCT01928459
BGJ398	FGFR1-3 TKI	2	Histologically confirmed GBM and/or other glioma subtypes with *FGFR1-TACC1, FGFR3-TACC3* fusion and/or activating mutation in *FGFR1, 2* or *3*	NCT01975701
BGJ 398	FGFR1-3 TKI	1	Advanced solid tumor Having alterations of the FGF-R pathway	NCT01697605
BLU-554	FGFR4 TKI	1	Refractory HCC or refractory advanced solid tumor other than HCC that has evidence of aberrant FGF19/FGFR4 pathway activity	NCT02508467
BT-701	Anti FGFR-3 antibody	2	Refractory UC of the bladder cancer or transitional cell carcinoma arising in another location of the urinary tract, including urethra, ureter, and renal pelvis with positive FGFR3 expression on IHC	NCT02401542
Debio 1347-101	FGFR1-3 inhibitor	1	Advanced solid malignancies, whose tumors have an alteration of the *FGFR 1, 2* or *3* genes, for whom standard treatment does not exist or is not indicated	NCT01948297
Dovitinib	Multikinase inhibitor including FGFR1-3	2	Refractory advanced/metastatic scirrhous gastric carcinoma	NCT01576380
Dovitinib	Multikinase inhibitor including FGFR1-3	2	*FGFR1* amplified and non-amplified metastatic HER2 negative breast cancer	NCT00958971
Dovitinib	Multikinase inhibitor including FGFR1-3	2	BCG refractory UC patients with tumor fibroblast growth factor receptor 3(FGFR3) mutations or over-expression	NCT01732107

Abbreviations: Fibroblast growth factor (FGF), fibroblast growth factor receptor (FGFR), not reported (NR), estrogen receptor positive (ER+), tyrosine kinase inhibitor (TKI), bacillus calmette-Guerin (BCG), small cell lung cancer (SCLC), Non-small cell lung cancer (NSCLC), squamous non-small cell lung cancer (sqNSCLC), pilot study (PS), glioblastoma multiforme (GBM), hepatocellular carcinoma (HCC), Malignant pleural mesothelioma (MPM), immunohistochemistry (IHC), urothelial carcinoma (UC). www.clinicaltrials.govaccessed on April 22^nd^ 2016.

**Table 5:** Ongoing selected clinical trials targeting the FGFR pathway in solid tumor and hematologic malignancies continued

Drug	Mechanism of action	Phase	Study population	Clinicaltrials.gov Identification
Dovitinib	Multikinase inhibitor including FGFR1-3	2	Either *FGFR2* mutated or wild-type advanced and/or metastatic endometrial cancer	NCT01379534
Dovitinib	Multikinase inhibitor including FGFR1-3	2	Refractory solid tumors with mutations or translocations of *FGFR, PDGFR, VEGF, cKIT, FLT3, CSFR1, Trk* and *RET*	NCT01831726
Dovitinib	Multikinase inhibitor including FGFR1-3	2	Advanced urothelial cancer with mutated or wild *FGFR3* mutated	NCT00790426
Divotinib	Multikinase inhibitor including FGFR1-3	2	Metastatic or unresectable gastric cancer harboring *FGFR2* amplification after failure of first or second sine chemotherapy	NCT01719549
Dovitinib	Multikinase inhibitor including FGFR1-3	NR	Refractory sqNSCLC with *FGFR* amplification (FISH > 5 copies of genes)	NCT01861197
Dovitinib	Multikinase inhibitor including FGFR1-3	2	Refractory progressive NSCLC and colorectal cancer status post antiangiogenic treatment	NCT01676714
Dovitinib	Multikinase inhibitor including FGFR1-3	PS	Refractory renal cell carcinoma	NCT01791387

Abbreviations: Fibroblast growth factor (FGF), fibroblast growth factor receptor (FGFR), not reported (NR), estrogen receptor positive (ER+), tyrosine kinase inhibitor (TKI), bacillus calmette-Guerin (BCG), small cell lung cancer (SCLC), Non-small cell lung cancer (NSCLC), squamous non-small cell lung cancer (sqNSCLC), pilot study (PS), glioblastoma multiforme (GBM), hepatocellular carcinoma (HCC), Malignant pleural mesothelioma (MPM), immunohistochemistry (IHC), urothelial carcinoma (UC).www.clinicaltrials.gov accessed on April 22^nd^ 2016.

**Table 6:** Ongoing selected clinical trials targeting the FGFR pathway in solid tumor and hematologic malignancies continued

Drug	Mechanism of action	Phase	Study population	Clinicaltrials.gov Identification
Dovitinib	Multikinase inhibitor including FGFR1-3	2	Refractory gastrointestinal stromal tumors	NCT01440959
E7090	FGF/FGFR pathway inhibitor	1	Refractory solid tumors dose expansion will enroll patients with tumor expressing genetic abnormality in FGF/FGFR pathway.	NCT02275910
FGF401	FGFR4-TKI	1/2	Hepatocellular carcinoma or solid malignancies characterized by positive FGFR4 and Klotho Berta (KLB) expression	NCT02325739
FPA144	FGFR2b antibody	1	Refractory solid tumors	NCT02318329
GSK3052230	FGF ligand trap (extra-cellular domain of FGFR1 fused with the Fc region of IgG1)	1b	Refractory progressive sqNSCLC with *FGFR1* gene amplification or MPM with measurable disease	NCT01868022
INCB054828	FGFR 1-3 TKI	1	Refractory solid tumors; on dose expansion subjects with sqNSCLC, gastric cancer, UC, endometrial cancer, multiple myeloma, or MPNs that have a tumor or malignancy that has been evaluated and confirmed to harbor genetic alterations in FGF or *FGFR* genes	NCT02393248
JNJ-42756493	Pan FGFR TKI	2	Metastatic or surgically unresectable UC that harbor specific FGFR genomic alterations	NCT02365597
JNJ-42756493	Pan FGFR TKI	1	Refractory HCC and for expansion phase participants must have *FGF19* amplification in addition	NCT02421185
JNJ-42756493	Pan FGFR TKI inhibitor	2a	Asian patients with advanced Non-small-cell lung cancer, urothelial cancer, gastric cancer, esophageal cancer or cholangiocarcinoma with *FGFR* gene mutation or translocation.	NCT02699606
JNJ-42756493	Pan FGFR TKI	1	Refractory solid tumors and lymphomas	NCT01962532

Abbreviations: Fibroblast growth factor (FGF), fibroblast growth factor receptor (FGFR), not reported (NR), estrogen receptor positive (ER+), tyrosine kinase inhibitor (TKI), bacillus calmette-Guerin (BCG), small cell lung cancer (SCLC), Non-small cell lung cancer (NSCLC), squamous non-small cell lung cancer (sqNSCLC), pilot study (PS), glioblastoma multiforme (GBM), hepatocellular carcinoma (HCC), Malignant pleural mesothelioma (MPM), immunohistochemistry (IHC), urothelial carcinoma (UC).www.clinicaltrials.gov accessed on April 22^nd^ 2016.

**Table 7:** Ongoing selected clinical trials targeting the FGFR pathway in solid tumor and hematologic malignancies continued

Drug	Mechanism of action	Phase	Study population	Clinicaltrials.gov Identification
JNJ-42756493	Pan FGFR TKI	1	Solid malignancy or lymphoma that is metastatic or unresectable, and for which standard curative treatment is no longer effective	NCT01703481
Lucitanib	VEGFR-FGFR Tyrosine Kinase Inhibitor	1/2a	Advanced solid tumors, relapsed or refractory to standard therapy. For the dose expansion, patients should have tumors bearing *FGFR1* or 11q 12-14 amplification, assessed by FISH or CGH array, or “sensitive” to antiangiogenic treatment	NCT01283945
Lucitanib	Multikinase inhibitor including FGFR	2	*FGFR1*-amplified or non-amplified ER+ metastatic breast cancer	NCT02053636
Lucitanib	Multikinase inhibitor including FGFR	2	Metastatic breast cancer	NCT02202746
Lucitanib	Multikinase inhibitor including FGFR	2	SCLC or NSCLC with tumor tissue based genetic alterations: *FGFR1, FGFR2, FGFR3, VEGFA*, or *PDGFRα* amplification; any *FGFR1, FGFR2*, or *FGFR3* gene fusion; *FGFR1, FGFR2*, or *FGFR3* activating mutation	NCT02109016
LY3076226,	FGFR3 Antibody-Drug Conjugate	1	Advanced refractory solid tumors with *FGFR3* alterations.	NCT02529553
Nintedanib	Multikinase inhibitor including FGFR	PS	Advanced refractory NSCLC with mutations, rearrangement and fusion involving *RET* oncogene, or abnormalities (non-synonymous SNV or amplification) in the nintedanib target genes *VEGFR1-3, TP53, PDGFR-A, PDGFR-B*, and *FGFR1-3*.	NCT02299141
Nintedanib	Multikinase inhibitor including FGFR	2	Refractory salivary gland tumors	NCT02558387
Nintedanib	Multikinase inhibitor including FGFR	2	Refractory small Cell Lung Cancer Patients Who Have Previously Benefited From First-line Platinum-based Chemotherapy	NCT02152059

Abbreviations: Fibroblast growth factor (FGF), fibroblast growth factor receptor (FGFR), not reported (NR), estrogen receptor positive (ER+), tyrosine kinase inhibitor (TKI), bacillus calmette-Guerin (BCG), small cell lung cancer (SCLC), Non-small cell lung cancer (NSCLC), squamous non-small cell lung cancer (sqNSCLC), pilot study (PS), glioblastoma multiforme (GBM), hepatocellular carcinoma (HCC), Malignant pleural mesothelioma (MPM), immunohistochemistry (IHC), urothelial carcinoma (UC).www.clinicaltrials.gov accessed on April 22^nd^ 2016.

**Table 8:** Ongoing selected clinical trials targeting the FGFR pathway in solid tumor and hematologic malignancies continued

Drug	Mechanism of action	Phase	Study population	Clinicaltrials.gov Identification
Nintedanib	Multikinase inhibitor including FGFR	PS	Refractory sqNSCLC	NCT01948141
Nintedanib	Multikinase inhibitor including FGFR	2	Advanced *FGFR3* mutated, *FGFR3* overexpressed, or FGFR3 wild type UC of urinary bladder, urethra, ureter, and renal pelvis and who have failed platinum-based chemotherapy	NCT02278978
Orantinib	Multikinase inhibitor including FGFR	1/2	Refractory HCC	NCT00784290
Pazopanib	Multikinase inhibitor including FGFR	PS	Refractory solid tumors *FGFR2* amplified and sensitive to pazopanib by Avatar scan	NCT02691767
Pazopanib	Multikinase inhibitor including FGFR	PS	Refractory solid tumors harboring *FGFR2* amplification or FGFR2 mutation	NCT02450136
PRN1371	FGFR1-4 TKI	1	Adults with advanced solid tumors	NCT02608125
Ponatinib	Multikinase inhibitor including pan-FGFR	2	Advanced biliary cancer with *FGFR2* fusion	NCT02265341
Ponatinib	Multikinase inhibitor including pan-FGFR	2	Refractory solid tumor or chronic hematologic solid malignancy with activating genomic alterations in *FGFR* (mutations, fusions or amplifications [> 6 copies]) or activating genomic alterations in *KIT*, platelet-derived growth factor receptor alpha [*PDGFRα*], ret proto-oncogene [*RET*], *ABL* proto-oncogene 1, non-receptor tyrosine kinase [*ABL1*] and fms-related tyrosine kinase 3 [*FLT3*]	NCT02272998

Abbreviations: Fibroblast growth factor (FGF), fibroblast growth factor receptor (FGFR), not reported (NR), estrogen receptor positive (ER+), tyrosine kinase inhibitor (TKI), bacillus calmette-Guerin (BCG), small cell lung cancer (SCLC), Non-small cell lung cancer (NSCLC), squamous non-small cell lung cancer (sqNSCLC), pilot study (PS), glioblastoma multiforme (GBM), hepatocellular carcinoma (HCC), Malignant pleural mesothelioma (MPM), immunohistochemistry (IHC), urothelial carcinoma (UC).www.clinicaltrials.gov accessed on April 22^nd^ 2016.

**Table 9:** Ongoing selected clinical trials targeting the FGFR pathway in solid tumor and hematologic malignancies continued

Drug	Mechanism of action	Phase	Study population	Clinicaltrials.gov Identification
Regorafenib	Multikinase inhibitor including FGFR	2	Refractory epithelial ovarian carcinoma (serous, clear cell, endometrioid, mucinous, mixed, carcinosarcoma and others), fallopian tube and primary peritoneal carcinoma)	NCT02736305
Sunitinib	Multikinase inhibitor including FGFR	PS	Refractory solid tumors harboring *RET* fusion positive or *FGFR2* amplification	NCT02450123
Sunitinib	Multikinase inhibitor including FGFR	PS	*RET* fusion positive or *FGFR2* fusion/other *FGFR* mutation Refractory solid tumor and/or specific sensitivity to Sunitinib by Avatar scan	NCT02691793
TAS120	FGFR TKI	1	Advanced metastatic solid tumors with or without abnormalities of FGF/FGFR who have failed all standard therapies or for whom standard therapy does not exist or multiple myeloma with amplification, mutation or translocation or other associated abnormalities of FGF/FGFR who have failed all standard therapies or for whom standard therapy does not exist	NCT02052778
U3-1784	FGFR4 monoclonal antibody	1	Refractory solid tumors	NCT02690350
XL228	Multi-targeted kinase inhibitor IGF-1R, Src, FGFR, and BCR-Abl	1	Refractory solid tumors, lymphoma, or multiple myeloma	NCT00526838

Abbreviations: Fibroblast growth factor (FGF), fibroblast growth factor receptor (FGFR), not reported (NR), estrogen receptor positive (ER+), tyrosine kinase inhibitor (TKI), bacillus calmette-Guerin (BCG), small cell lung cancer (SCLC), Non-small cell lung cancer (NSCLC), squamous non-small cell lung cancer (sqNSCLC), pilot study (PS), glioblastoma multiforme (GBM), hepatocellular carcinoma (HCC), Malignant pleural mesothelioma (MPM), immunohistochemistry (IHC), urothelial carcinoma (UC).www.clinicaltrials.gov accessed on April 22^nd^ 2016.

## DISCUSSION

In the recent years the field of medical oncology has witnessed unprecedented advances in the understanding of cancer biology. FGF/FGFR pathway is a thriving area of targeted drug development in a wide array of tumors (Tables 3, 4, 5, 6, 7, 8, 9). *FGFR3-TACC3* was first described in 2012 in two patients with GBM. [13] Since then *FGFR3-TACC3* fusion has been reported in numerous solid tumors including urothelial carcinoma, NSCLC, thyroid, and cervical carcinoma (Table 2). [12] In addition dataset analysis through bioinformatics, namely the TCGA dataset, yield other rarer instances in which this aberration can be found such as prostate cancer, head and neck cancer, and kidney papillary cancer. [14]

The clinical relevance of *FGFR3-TACC3* has been highlighted by 3 out of 4 partial responses among patients with tumors harboring *FGFR3-TACC3* fusions treated with FGFR inhibitor JNJ-42756493. [10] In agreement with preclinical results, these results suggest that *FGFR3-TACC3* fusion is indeed an actionable therapeutic target. Furthermore taking into account the obvious limitation of the sparse clinical data thus far tumors harboring *FGFR3-TACC3* fusions seem to be more sensitive FGFR targeted therapies when compared to other *FGFR* aberrations. This is particularly important, as there are limited treatment options for patients with aggressive tumors in which *FGFR3-TACC3* fusions have been described such as GBM and bladder cancer. Future studies should take into account that *FGFR3-TACC3* fusion has shown positive correlations with PI3K/AKT/mTOR pathway, cell cycle control (*CDK4*, *CDK2*, and *CCND1*), and *MDM2* aberrations (Table 1). [23, 36, 39] The corollary to these concomitant findings is that one could hypothesize that the combined approach targeting *FGFR3-TACC3* and its downstream oncogenic proteins may further enhance the efficacy of FGFR aberrant targeted therapy. Notwithstanding its rarity, *FGFR3-TACC3* fusions are present in wide array of solid tumor types and its analysis should be an integral part of screening procedures in FGFR targeted trials in solid tumors.

Elucidation of the potential interaction between *FGFR* and its fusions with the immune system is also warranted. Pre-clinical data suggest that *FGFR3* mutations are exclusive to non-inflamed bladder cancers (~ 30% of tumors), a subgroup characterized by absence of CD8 tumor infiltrating lymphocytes (CD8 TILs), worsen prognosis, and lower chance of responding to PD-L1 blockade. [40–42] The exclusion of CD8 TILs from the tumor microenvironment limits the benefit from immunotherapies in melanoma and might carry similar relevance to bladder cancer. [42] Nevertheless, these preliminary results highlight a possible link between FGFR pathway aberrations and immune modulation of tumor microenvironment that could be explored therapeutically.
